# The Use of Fluoroquinolones for Tuberculosis in Victoria between 2011 and 2016

**DOI:** 10.1155/2018/6860479

**Published:** 2018-07-02

**Authors:** Hugh Murray, Ee Laine Tay, Sarah Dinh, Helen Matthews, Alan Street, Justin T. Denholm

**Affiliations:** ^1^Victorian Tuberculosis Program, The Peter Doherty Institute for Infection and Immunity, Australia; ^2^Department of Health and Human Services, Victoria, Australia; ^3^Department of Pharmacy, Royal Melbourne Hospital, Australia; ^4^Victorian Infectious Diseases Service, Royal Melbourne Hospital, Australia; ^5^Department of Microbiology and Immunology, University of Melbourne, Australia

## Abstract

*Setting. *Tuberculosis treatment requires long regimens with multiple antibiotics and is complicated by antibiotic resistance and intolerance. Fluoroquinolones were introduced for the treatment of multidrug resistant TB and have become a vital part of therapy.* Objective. *Reviewing the indications for fluoroquinolones use in the treatment of active TB in Victoria, Australia.* Design. *This was a retrospective case-control study of Victorian patients prescribed fluoroquinolones for active tuberculosis, from January 2011 to December 2016. Indications for fluoroquinolone use were extracted from an existing public health database.* Results. *There were 2268 patients notified to have tuberculosis in Victoria between 2011 and 2016, 276 (12.2%) of whom received a fluoroquinolone. The indications were substitution when intolerance was present (33.3%) or anticipated (21.0%), proven drug resistance (22.5%), suspected drug resistance (13.0%), and site of disease (10.1%). Where fluoroquinolones were prescribed for suspected drug resistance, only a minority of isolates (13%) had resistance confirmed.* Conclusion. *Fluoroquinolones were most commonly used as replacement for first-line therapy related to adverse effects, when either present or anticipated. Where fluoroquinolones were prescribed for suspected drug resistance, only a minority of isolates ultimately had resistance confirmed. These findings reinforce the importance of fluoroquinolones in TB therapy and the need for ongoing pharmacovigilance to ensure appropriate use.

## 1. Introduction

Tuberculosis (TB) is an ongoing public health challenge with an estimated 10.4 million new TB cases worldwide in 2015 [1]. Prolonged courses of therapy with multiple antibiotics are required, and complications such as antibiotic resistance and drug intolerance may require alternatives to standard first-line medications. Fluoroquinolones are a key component of the treatment of multidrug resistant TB (MDR-TB), which is by definition resistant to both isoniazid and rifampicin [2]. This class of drugs may also be used for replacing first-line medication in the setting of drug intolerance, and there is theoretical and some early clinical evidence of possible increased efficacy in TB meningitis and ocular TB [3–5]. In addition, there has been interest in recent years in the use of later-generation fluoroquinolones as part of first-line therapy, although large randomised controlled trials failed to show noninferiority of fluoroquinolone containing and shortened regimens when compared to the standard treatment regimen [6–8].

The World Health Organisation (WHO) recommends the use of a fluoroquinolones in first-line therapy for MDR-TB [9]. The Centre for Disease Control (CDC), American Thoracic Society, and Infectious Diseases Society of America also recommend FQN as an alternative to first-line agents if hepatotoxicity occurs [10].

Given the variety of ways in which fluoroquinolones may be used in TB therapy, understanding the circumstances and justification for their use by clinicians is of interest. However, while many recommendations for their use exist, few real-world data on clinical application have been available.

## 2. Study Population and Methods

This was a retrospective case-control study of cases with active TB notified in Victoria, Australia, from the 1^st^ of January 2011 to the 31^st^ of December 2016, in which the use and indications for fluoroquinolones were described.

The State of Victoria has a population of approximately 5.7 million, with an incidence of TB of 6.7 per 100,000 in 2013 [11]. The Victorian Tuberculosis Program is the statewide authority responsible for TB public health management, including medication supply and contact tracing [12]. Notification of all cases of TB is compulsory in both public and private healthcare settings, with case management and outcome records maintained in a central existing electronic public health record system [13]. This system also includes laboratory diagnostic records, including molecular and phenotypic drug susceptibility testing results. Data for this review, including demographic and clinical details, isolate susceptibility patterns, and medications prescribed were extracted from this database. Cases were defined as any patients within the study period who received ≥1 dose of a fluoroquinolone for the treatment of active TB, with all other cases during the study period used as controls.

Following extraction, patient case notes were manually reviewed for fluoroquinolone indications. As indications were not prospectively recorded systematically, cases where indication was not explicitly recorded were determined by consensus after clinician review of all documented case characteristics, for example, site of disease, adverse effects, or overall regimen. Indications were categorized into mutually exclusive groups for the purpose of statistical analysis. The groups were as follows: known drug resistance (subcategorized into MDR-TB and other patterns of drug resistance); suspected drug resistance; drug intolerance; adverse effect avoidance; site of disease; or shortening of the treatment regimen. Here, adverse effect avoidance is the substitution of a first-line agent for a fluoroquinolone due to a predicted interaction between its side effects and the patient's comorbidities, for example, substituting moxifloxacin for ethambutol when existing visual impairment is present. These categories were chosen based on review of the literature and discussion with expert TB clinicians. Where more than one reason may have been present, explicitly stated reasons for use were preferred for assigning a primary category. Fluoroquinolone resistance patterns were also determined for the MDR-TB cases found.

No national guidelines are in place for the use of fluoroquinolones in TB for Australian clinicians. In Victoria, the Department of Health and Human Services contemporaneously recommended the use of fluoroquinolones in place of ethambutol “in patients with renal failure, significant preexisting ocular disease or where ocular toxicity cannot be monitored” and as part of the treatment of MDR-TB. No specific recommendations were in place for other indications, but it is expected that clinicians were aware of relevant international recommendations, including the relevant IDSA and WHO guidelines, during this period.

Ethics approval was obtained from the Melbourne Health Human Research Ethics Committee. Microsoft Excel 2010 and STATA (version 14.0, College Station, Texas) were used for statistical analysis. Univariate logistic regression analysis was performed to identify variables that were associated with fluoroquinolone use with p value of <0.05 considered significant. Multivariate logistic regression analysis was conducted on variables prespecified on the basis of literature review, with additional variables included where p ≤0.20 on univariate analysis. Analysis for interaction between relapse status and resistance pattern was performed.

## 3. Results

There were 2268 notifications of active TB in Victoria between 2011 and 2016 and 281 (12.2%) received a fluoroquinolone during TB therapy. Of these, three were prescribed ciprofloxacin for other conditions, and 278 received moxifloxacin. In two of those cases, moxifloxacin was deemed to have been administered for conditions other than TB, so the remainder of this analysis was based on the 276 (12.2%) who were prescribed moxifloxacin for the treatment of active TB. Levofloxacin was temporarily substituted for moxifloxacin in one patient due to concern about potential interaction between moxifloxacin and bedaquiline. The characteristics of these cases are described in Table 1. There was no significant change in the amount of moxifloxacin used over the time period.

**Figure 1:**
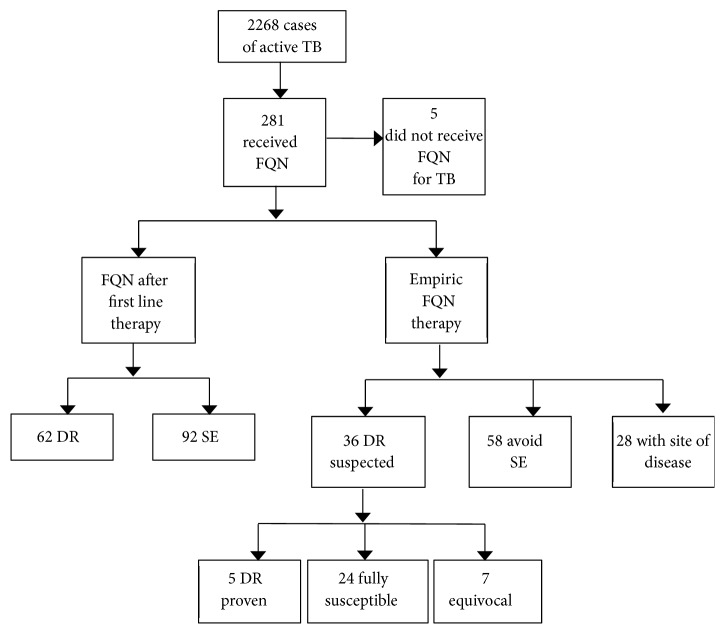
Indications for use of moxifloxacin for tuberculosis between 2011 and 2016. DR: resistance, SR: suspected resistance, SE: side effects, and FQN: fluoroquinolone.

The indications for moxifloxacin use are presented in Figure 1. The most common indications involved either cases experiencing adverse effects (92; 33%) or clinicians avoiding a medication where a high risk of adverse effects was predicted (58; 21%). Due to limitations with the data, a quantitative analysis of side effects was not possible; however broadly they were predominantly hepatotoxic and visual and visual and renal for side effects experienced and predicted, respectively. Drug resistance was present in 62 (22%) cases, with a further 36 (13%) prescribed moxifloxacin due to clinician concerns regarding possible resistance. In the latter scenario it was predominantly prescribed as a single addition to the four first-line agents. The sites of disease for which moxifloxacin was specifically chosen included meningeal (11), ocular (7), other central nervous system (CNS) (4), and other sites (6; lymph node, bone and joint, skin, gastrointestinal, and disseminated).

**Figure 2:**
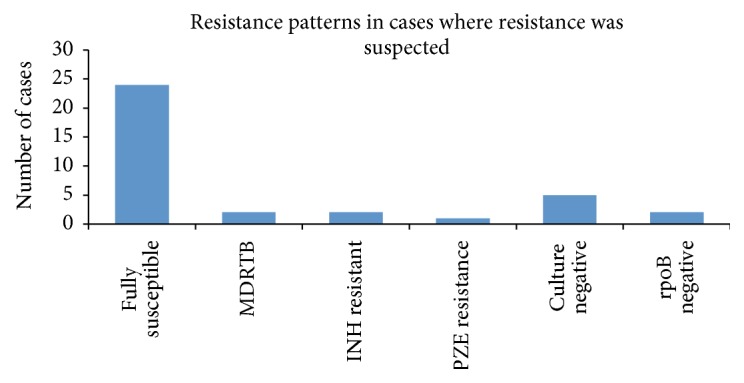
Resultant resistance patterns in 36 cases where clinicians suspected antibiotic resistance prior to sensitivity results being available. rpoB: rapid test for rifampicin resistance, INH: isoniazide, and PZE: pyrazinamide.

Of the 36 cases where moxifloxacin was used because of concerns about possible drug resistance, susceptibility results are shown in Figure 2. Two-thirds of cases were fully susceptible (24), with two cases being MDR-TB, three with other resistance patterns, and seven being culture negative. In those 24 cases that were fully sensitive, the original indications for choosing moxifloxacin were previous TB treatment in 15 cases, patient originating from a TB endemic country in seven cases, and exposure to a resistant case in one case and one with an unclear reason.

All confirmed MDR-TB cases during the study period (39) received moxifloxacin. Of these, 30 were sensitive to ofloxacin (76.9%), four were ofloxacin resistant with moxifloxacin sensitivity unavailable (10.3%), and two were resistant to both (5.1%), and for three no quinolone sensitivities were performed (7.7%).

Multivariate analysis of moxifloxacin use is presented in Table 2. The strongest associations were observed where drug resistance and meningeal and ocular TB were present, with MDR-TB not separately included due to 100% concordance. No interaction was found between antibiotic resistance and relapsing disease.

## 4. Discussion

Fluoroquinolones are critical therapeutic options for TB, and appropriate stewardship of use is important for ensuring their continued effectiveness. In this study, we found that moxifloxacin was most commonly used to ameliorate the adverse effects of first-line therapy, both when they were predicted and after they occurred. The use of moxifloxacin for treating confirmed drug resistant cases of TB was anticipated and confirmed. This review also identified inappropriate, singular addition of moxifloxacin to the first-line regimen when drug resistance was suspected.

The majority of MDR-TB cases occurring in Victoria during the study period were confirmed as sensitive to moxifloxacin through ofloxacin susceptibility testing [14]. Specific moxifloxacin sensitivity testing was not introduced until 2015 in Victoria. After this time there were two cases of confirmed moxifloxacin resistance which highlights the need for resistance testing, particularly as moxifloxacin monotherapy is used as preventative therapy for contacts in this setting [15].

When moxifloxacin was commenced on the basis of suspected drug resistance, two-thirds of cases were ultimately found to be fully susceptible. In 63% of these sensitive cases clinicians originally suspected resistance due to having received treatment previously, while in 29% it was suspected due to their origin from a TB endemic country. The WHO estimated that worldwide in 2015 21% of relapsing disease in patients previously treated for TB was MDR-TB or rifampicin resistant TB. Of the MDR-TB cases between 1998 and 2012 in Australia, 25.8% were in previously diagnosed and treated patients [1, 16]. However, traditional risk factors (i.e., history of TB treatment and poor adherence to treatment) for predicting drug resistance are increasingly recognized to be misleading, with a high proportion of MDR-TB acquired through transmission rather than amplification of resistance. In cases where drug resistance is suspected, rapid genotypic evaluation should be conducted, and where empiric therapy is required a full MDR-TB treatment regimen should be used, rather than singular addition of a fluoroquinolone to a first-line regimen.

The use of moxifloxacin for particular sites of disease was predominantly seen in cases of CNS TB (including meningitis) and ocular TB. There is currently no clinical evidence for the addition of a fluoroquinolone in the treatment of TB meningitis with two trials not demonstrating a survival benefit [4, 17]. However, there is evidence that moxifloxacin better penetrates the cerebrospinal fluid than ethambutol, particularly in noninflamed meninges, and given the severity of CNS TB we would consider this an appropriate indication [3, 18, 19].

This study was limited by its retrospective nature and primary reliance on nursing case management records in which indications for moxifloxacin use were occasionally not explicitly mentioned. However, our use of single statewide database and centralized public health and laboratory program has allowed a more complete health service level review than would have been possible otherwise. Future stewardship initiatives will benefit from prospective data collection regarding medication use and allow for interventions to reduce inappropriate use of fluoroquinolones and other critical TB medications.

## 5. Conclusions

This study has described the use of fluoroquinolones for treatment of tuberculosis between 2011 and 2016. In our setting, we found that moxifloxacin was the predominant fluoroquinolone used, particularly in the setting of intolerance to first-line medications and drug resistant disease but also when resistance is suspected and for particular sites of disease. These findings, particularly the identification of inappropriate fluoroquinolone use for cases where drug resistance was suspected, will allow consideration of further antibiotics stewardship initiatives to promote optimal use of TB medications.

## Figures and Tables

**Table 1:** Characteristics of cases who received moxifloxacin versus those who did not receive moxifloxacin.

Factors	Moxi	(%)	Non-Moxi	(%)
Number	276		1992	
Age	44.78		38.5	
Sex (male)	150	(54.3)	1129	(56.7)

Site of disease				
Pulmonary	165	59.8	1182	59.3
Meningeal	12	4.3	9	0.5
Central Nervous System	2	0.7	4	0.2
Ocular	10	3.6	12	0.6
Other extra-pulmonary	87	31.5	786	39.4

Country of origin				
Australian	22	8.0	219	11.0
Overseas	254	92.0	1773	89.0

HIV status				
Positive	5	1.8	32	1.6
Negative	67	24.3	328	16.5
Unknown	204	73.9	1632	81.9

Diagnosis method				
Culture	217	78.6	1521	76.3
Histological	13	4.7	161	8.1
PCR/NAT	19	6.9	116	5.8
Radiological	9	3.3	126	6.3
Clinical	17	6.2	58	2.9
Microscopic Examination	1	0.4	10	0.5

Sensitivities - Of culture positive cases				
Fully sensitive	148	68.2	1410	92.6
MDRTB	39	18.0	0	0.0
XDRTB	2	0.9	0	0.0
Other resistance patterns	26	12.0	103	6.8
Isoniazid Resistance	24	92.3	97	94.2
Rifampicin resistance	1	3.8	0	0.0
Pyrazinamide resistance	1	3.8	2	1.9
Ethambutol resistance	0	0.0	3	2.9
Other agents	0	0.0	1	1.0
Sensitivity results pending	2	0.9	9	0.6

**Table 2:** Univariate and multivariate analysis of factors associated with moxifloxacin use in all TB cases over the 2011-16. Significant results in the multivariate analysis are highlighted in **bold**.

Patient demographics, diagnosis, and microbiological factors	Use of moxifloxacin	
	Univariate analysis (OR; 95% CI; p-value)	Multivariate analysis (OR; 95% CI; p-value)
Age(>65 years)	**1.64** (1.19-2.27) p = 0.002	**1.70** (1.19-2.41) p = 0.003
Sex	0.87 (0.68-1.12) p = 0.28	-
Overseas born	1.42 (0.90-2.24) p = 0.13	1.2 (0.74-1.96) p = 0.47
Relapsing disease	**6.35** (4.17-9.67) p < 0.001	**5.59** (3.55-8.80) p < 0.001
All non-pulmonary disease	0.98 (0.76-1.27) p = 0.89	-
Meningeal/Central nervous system TB	**6.88** (3.34-14.19) p < 0.001	**10.55** (4.97-22.38) p < 0.001
Ocular TB	**6.12** (2.60-14.39) p < 0.001	**9.88** (3.91-24.96) p < 0.001
HIV positive	1.13 (0.44-2.93) p = 0.8	-
Non-culture confirmed	0.88 (0.65-1.19) p = 0.40	-
Antibiotic resistance	**6.40** (4.52-9.08) p < 0.001	**6.36** (4.42-9.20) p < 0.001

## Data Availability

The data used here contains confidential patient information; therefore access to it would require approval from Melbourne Health Human Research Ethics Committee.

## Abbreviations

CNS: Central nervous system
ETH: Ethambutol
INH: Isoniazid
MDR-TB: Multidrug resistant tuberculosis
Moxi: Moxifloxacin
NAT: Nucleic acid testing
PCR: Polymerase chain reaction
PHESS: Public Health Event Surveillance System
PZE: Pyrazinamide
RIF: Rifampicin
TB: Tuberculosis
XDR-TB: Extensively drug resistant tuberculosis.
